# New Drugs, Appliances, and Things Medical

**Published:** 1890-05-17

**Authors:** 


### MEDICINAL SOAP.

### NEW DRUGS, APPLIANCES, AND THINGS MEDICAL.

[All preparations, appliances, novelties, etc., of which a notice is desired, should be sent to The Editor, The Lodge, Porchester Square, W.]

### "VINOLIA" TOILET SOAP.

Messrs. Blondeau and Co., Ryland Road, Kentish Town, have forwarded to us samples of the above. It proves to be a pure soap with no excess of alkali, and containing an excess of fatty material, forming a most excellent specimen of a "super fatted" soap. We have used this firm's manufactures for the last year or two, and have found them satisfactory. The soap is useful for all delicate skins, whether there be actual disease or not. It is particularly suited for the nursery, as anything more irritating than ordinary soap to the skin of infants and young children can hardly be found. The specimens of toilet soap are pleasantly scented and neat in appearance.

